# Evidence of a 2D Electron Gas in a Single‐Unit‐Cell of Anatase TiO_2_ (001)

**DOI:** 10.1002/advs.202105114

**Published:** 2022-04-05

**Authors:** Alessandro Troglia, Chiara Bigi, Ivana Vobornik, Jun Fujii, Daniel Knez, Regina Ciancio, Goran Dražić, Marius Fuchs, Domenico Di Sante, Giorgio Sangiovanni, Giorgio Rossi, Pasquale Orgiani, Giancarlo Panaccione

**Affiliations:** ^1^ Istituto Officina dei Materiali (IOM)‐CNR Laboratorio TASC in Area Science Park, S.S. 14 Km 163.5 Trieste 34149 Italy; ^2^ Dipartimento di Fisica Universitá di Milano Via Celoria 16 Milano 20133 Italy; ^3^ Department of Materials Chemistry National Institute of Chemistry Hajdrihova 19 Ljubljana 1001 Slovenia; ^4^ Institut für Theoretische Physik und Astrophysik and Würzburg‐Dresden Cluster of Excellence ct.qmat Universität Würzburg Würzburg 97074 Germany; ^5^ Department of Physics and Astronomy University of Bologna Bologna 40127 Italy; ^6^ Center for Computational Quantum Physics Flatiron Institute 162 5th Avenue New York NY 10010 USA

**Keywords:** 2D electron gas, anatase, angle‐resolved photo‐electron spectroscopy, ultra‐thin oxides

## Abstract

The formation and the evolution of electronic metallic states localized at the surface, commonly termed 2D electron gas (2DEG), represents a peculiar phenomenon occurring at the surface and interface of many transition metal oxides (TMO). Among TMO, titanium dioxide (TiO_2_), particularly in its anatase polymorph, stands as a prototypical system for the development of novel applications related to renewable energy, devices and sensors, where understanding the carrier dynamics is of utmost importance. In this study, angle‐resolved photo‐electron spectroscopy (ARPES) and X‐ray absorption spectroscopy (XAS) are used, supported by density functional theory (DFT), to follow the formation and the evolution of the 2DEG in TiO_2_ thin films. Unlike other TMO systems, it is revealed that, once the anatase fingerprint is present, the 2DEG in TiO_2_ is robust and stable down to a single‐unit‐cell, and that the electron filling of the 2DEG increases with thickness and eventually saturates. These results prove that no critical thickness triggers the occurrence of the 2DEG in anatase TiO_2_ and give insight in formation mechanism of electronic states at the surface of TMO.

## Graphical abstract

This paper reports the evolution of a 2D electron gas (2DEG) in anatase TiO_2_ thin films as a function of thickness. The investigation reveals that, once the anatase fingerprint is present, the 2DEG in TiO_2_ is robust and stable down to a single‐unit‐cell. The results prove that no critical thickness triggers the occurrence of the 2DEG in anatase TiO_2_.

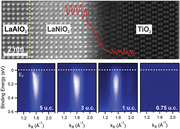

## 1 Introduction

Anatase titanium dioxide (TiO_2_) is a system that raises high expectations as a strategic material for novel devices such as memristors, transparent conductive oxides, and solar cells.^[^ ^1^ ^]^ These devices strongly rely on the conductive behavior of the surface and it is therefore crucial to better understand and control its properties. Like many similar systems such as SrTiO_3_,^[^ ^2^, ^3^ ^]^ KTaO_3_^[^ ^4^, ^5^ ^]^ and other titanates,^[^ ^6^, ^7^ ^]^ anatase TiO_2_ displays a highly conductive metallic state at the surface, commonly defined as 2D electron gas (2DEG).^[^ ^8^, ^9^, ^10^, ^11^ ^]^ Nevertheless, despite the enormous research activity in the past few years, the physical mechanisms responsible for the formation of a 2DEG at the surface of insulating oxides or at their interface still remains one of the most debated issues in this field. For instance, in the case of SrTiO_3_‐based interfaces,^[^ ^12^, ^13^ ^]^ many reports have indeed pointed toward the building of an internal electrical potential as mandatory condition to drive the formation of the 2DEG.^[^ ^13^, ^14^ ^]^ Such a scenario appears to be confirmed by the existence of a threshold thickness of the oxide layers needed to show the highly conductive state.^[^ ^15^, ^16^, ^17^, ^18^, ^19^ ^]^ Extrinsic mechanisms, attributed to structural imperfections (mainly to oxygen vacancies) have been also suggested as responsible for the formation of the 2DEG,^[^ ^20^, ^21^, ^22^ ^]^ in this case with no need of a critical thickness, leaving the origin of the 2DEG an open question.

In this framework, although the formation of a 2DEG in anatase TiO_2_ has been mainly ascribed to oxygen vacancies,^[^ ^23^ ^]^ there is no conclusive evidence for a critical thickness not being one of the requirements to drive the formation of the 2DEG, which is instead a mandatory condition for similar systems such as the LaAlO_3_/SrTiO_3_ hetero‐interface.^[^ ^15^ ^]^ Moreover, the dimensionality of the 2DEG, particularly in anatase TiO_2_, is still debated: Moser et al.^[^ ^8^ ^]^ report a 3D character deduced from ARPES measurements as a function of perpendicular momentum, while in contrast Rödel et al.^[^ ^9^ ^]^ and Wang et al.^[^ ^10^ ^]^ report a highly 2D nature in similar measurements. Recently, experimental and theoretical reports suggest that the delocalized oxygen vacancies in the subsurface region are responsible for the formation of the 2DEG,^[^ ^23^ ^]^ supporting a 2D character of the dispersive features. On the other hand, Ma et al.^[^ ^24^ ^]^ propose a coexistence of both 2D and 3D characters, similar to what has been observed in other oxides.^[^ ^25^, ^26^ ^]^ In this framework, the only way to provide conclusive evidence regarding the nature and the dimensionality of the 2DEG is to check the evolution of the 2DEG versus material thickness in carefully controlled thin films.

Here we investigated the formation and the evolution of the 2DEG in anatase TiO_2_ down to the single‐unit‐cell and sub‐unit‐cell thickness by means of X‐ray based electron spectroscopies. Films were grown in situ by pulsed laser deposition (PLD) and subsequently transferred across the continuous ultra‐high vacuum manifold to the synchrotron radiation spectrometers of the NFFA‐APE beamlines at the Elettra facility in Trieste.^[^ ^11^, ^23^, ^27^ ^]^ Angular‐resolved photo‐emission spectroscopy (ARPES), X‐ray absorption spectroscopy (XAS) and X‐ray photo‐emission spectroscopy (XPS) allow to detect the development of 2DEG along with that of the spectroscopic fingerprint of anatase. We observe that as soon as a single‐unit‐cell of anatase TiO_2_ is formed, the 2DEG is also observed. ARPES measurements as a function of film thickness, supported by DFT calculations, show that the electron‐filling of the 2DEG increases with increasing thickness and eventually saturates. Our results indicate that no critical thickness is required to stabilize the 2DEG at the anatase TiO_2_ surface, implying that the anatase 2DEG has a different nature than the 2DEG states found in other TMO^[^ ^5^, ^28^, ^29^, ^30^, ^31^ ^]^ and in particular at the LaAlO_3_/SrTiO_3_ hetero‐interface, where a critical thickness of 1.5 nm (4 u.c.) is needed to establish the 2DEG.^[^ ^15^ ^]^

## 2 Results and Discussion

**Figure 1:**
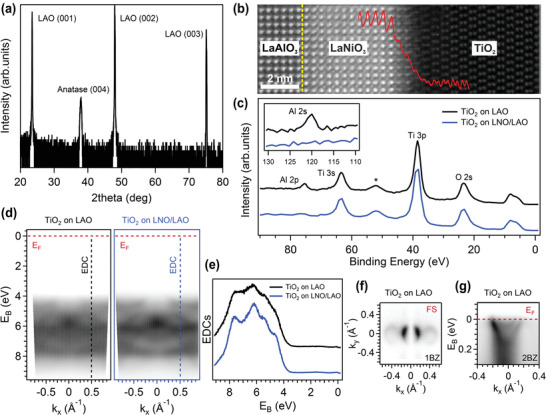
a) Symmetrical θ–2θ high‐resolution XRD scan of a thick (i.e., more than 15 nm) sample of anatase TiO_2_ grown onto LAO (001) substrate. b) High‐resolution cross‐sectional STEM HAADF analysis showing film, buffer layer, and substrate with superimposed intensity profile over the LNO/TiO_2_ interface (red curve). The position of the LAO/LNO interface is highlighted by a yellow dashed line. c) XPS spectra of anatase TiO_2_ thin films grown onto LAO (001) substrate with and without a LNO buffer layer (blue/black curves respectively), acquired with a photon energy hν = 900 eV. d) ARPES band dispersion spectra of anatase TiO_2_ grown onto LAO (001) substrate with and without a LNO buffer layer. The spectra have been acquired at hν = 46 eV and horizontal polarization. e) EDCs extracted at *k*_x_=0.5 Å^−1^ (dashed lines in panel (d)). f) ARPES map in the first Brillouin zone (1 BZ) of anatase TiO_2_ grown onto LAO (001) substrate acquired at Fermi energy with a photon energy hν = 46 eV and horizontal polarization. g) ARPES spectrum of the metallic state of anatase TiO_2_ grown onto LAO (001) substrate acquired with a photon energy hν = 46 eV and horizontal polarization around the $\bar{\Gamma }$ point of the second Brillouin zone (2 BZ).

High‐quality anatase (001)‐TiO_2_ thin films have been epitaxially grown by means of pulsed laser deposition (PLD) on LaAlO_3_ (LAO) substrates. Details about the optimal deposition parameters can be found elsewhere.^[^ ^11^, ^23^ ^]^ The high‐resolution symmetrical θ–2θ scans reported in **Figure** **1**a prove that our films are preferentially oriented with the TiO_2_ *c*‐axis parallel to the [001] crystallographic direction of the substrate. Only the (00*l*) diffraction peaks of the films are detected in the diffraction pattern, with no trace of impurity phases. The very low lattice mismatch imposed by the LAO substrate (<−0.1%) allows the (001) anatase TiO_2_ thin films to grow fully epitaxially with the in‐plane lattice parameters of the LAO substrate as shown by reciprocal space map around the (113)‐LAO and (116)‐TiO_2_ asymmetric reflections reported in Figure S1, Supporting Information, showing a perfect alignment of the diffraction peaks along the in‐plane direction Q_*x*_. Given the very low lattice mismatch, the films are considered unstrained. Consistently with this assumption, the out‐of‐plane lattice parameter has been measured to be 0.948 ± 0.001 nm, in very good agreement with the expected *c*‐axis parameter of the relaxed TiO_2_ anatase.^[^ ^32^ ^]^

Given that the primary scope of our work is to investigate the evolution of the 2DEG in extremely thin TiO_2_ anatase films, the optimal quality of the substrate‐film interface is crucial. Previous high‐resolution transmission electron microscopy (HR‐TEM) studies have shown that the crystal structure of the TiO_2_ films is strongly influenced by the bare substrate and by the growth protocol.^[^ ^33^, ^34^, ^35^ ^]^ Moreover, XPS results show Al intercalation in TiO_2_ films grown on bare LAO:^[^ ^11^ ^]^ even though Al has been proven to be homogeneously distributed over the entire film rather than localized at the interface, we cannot exclude an influence on the electronic properties of the 2DEG in ultra‐thin films. Therefore, the use of an Al‐free buffer layer for our thinnest anatase films has been preferred. The optimal buffer layer must fulfill two requirements: i) to prevent the Al interdiffusion from the substrate and ii) maintain the high structural perfection of the LAO buffer layers. We found LaNiO_3_ (LNO) perovskite compound as the ideal candidate. LNO preserves the in‐plane lattice matching between TiO_2_ thin films and LAO substrates thanks to the very small (i.e., 1%) lattice mismatch.

The high‐quality of the films and interfaces is also visible from cross‐sectional TEM analysis in Figure 1b. The image depicts an atomically resolved scanning transmission electron microscopy (STEM) high‐angle annular dark‐field (HAADF) micrograph showing the interface region between substrate, buffer, and film. From this image we see that the interface between substrate and buffer is atomically sharp and that film, buffer, and substrate indeed fully match in‐plane. The slightly blurred transition region between buffer and film is explained by the slight inherent surface roughness of the PLD grown buffer layer, which does not affect the continuity and quality of the film. The typical dumbbell structure of the anatase Ti sublattice can be discerned across the LNO/TiO_2_ interfacial region, with no presence of bare extra‐layers at the film/substrate interface. This is further emphasized by the intensity profile superimposed to the image, which shows the expected LAO/LNO/TiO_2_ atomic stacking sequence across the interfaces.

The insertion of a LNO buffer layer ≈5 nm thick (see Figure 1b) allowed the suppression of the Al intercalation from the LAO substrate into the film, as confirmed by XPS results displayed in Figure 1c. One observes the disappearance of the Al peaks (2s and 2p) in TiO_2_ thin films when grown on LNO‐buffered LAO substrate, which is consistent with other works reported in the literature.^[^ ^36^, ^37^, ^38^ ^]^ We stress here that the suppression of the Al interdiffusion is not ascribable to the thickness of the TiO_2_ film; in fact, hard X‐ray PES measurements, with probing depth in the range of 8–10 nm,^[^ ^39^, ^40^, ^41^ ^]^ show Al homogeneously distributed over the entire film of a 20 nm thick anatase TiO_2_ film.^[^ ^11^ ^]^ Moreover, the metallic LNO character provides better sample grounding and minimize charging effects. In fact, the typical resistivity value at room temperature measured in a four‐probe van der Pauw configuration^[^ ^42^ ^]^ was ≈1 mΩ cm, that is only one order of magnitude higher than the best LNO metallic thin film reported in literature.^[^ ^37^, ^38^ ^]^ Moreover, the insertion of a metallic LNO buffer layer improves significantly the quality of the anatase films, as shown by the ARPES spectra presented in Figure 1d,e, where we compare the band dispersion of TiO_2_ on bare LAO (left) and on LNO‐buffered LAO (right) substrates and the corresponding EDC curves extracted at *k*_x_ = 0.5 Å^−1^ (marked with dashed lines in panel (d)). Both samples display the same spectral features in the valence band and at the Fermi level. However, the bands appear sharper and better defined when TiO_2_ is grown on the LNO‐buffered substrate, as clearly visible from the EDC curves reported in panel (e) of Figure 1.

Panels (f) and (g) of Figure 1 show that the 2DEG state is characterized by a parabolic dispersion and circular Fermi contours. The replicas of the 2DEG states along the *k*_*x*_ and *k*_*y*_ directions mimic the (1×4)–(4×1) reconstruction of the anatase (001) surface.^[^ ^10^, ^43^, ^44^ ^]^ The strong suppression of the photoemitted intensity along the *k*_x_ = 0 Å^−1^ direction arises from matrix element effects and it is consistent with the d_*xy*_ Ti 3d orbital character in anatase^[^ ^8^, ^9^, ^10^, ^23^ ^]^ and other Ti‐based oxides.^[^ ^2^, ^3^ ^]^

Figure 1g shows the parabolic dispersion of the 2DEG in the second Brillouin zone (BZ), where matrix element effects do not cause intensity suppression except for a left‐to‐right asymmetry in the photoemitted intensity distribution.^[^ ^2^, ^8^, ^23^ ^]^ Two electronic states with parabolic dispersion are visible: an outer parabola and a faint quantized subband related to the confinement potential at the surface, in agreement with previous measurements.^[^ ^23^ ^]^ The electron density (*n*_2D_ ≈ 4× 10^13^ cm^−2^) extracted from the Fermi momenta and the Luttinger theorem for a spin degenerate, 2D state^[^ ^45^ ^]^ is consistent with other works on high carrier's concentration transition metal oxide systems.^[^ ^8^, ^10^, ^44^, ^46^ ^]^ In particular, no clear Fröhlich polaron satellites are detected in our spectra at the experimental temperature (78 K), consistent with the increased screening in the sample for high carrier's concentrations.^[^ ^8^, ^47^, ^48^ ^]^ However, the signature of the electron–phonon coupling is visible in Figure 1g, where the ARPES spectral intensity broadens and extends well below the parabolic dispersion. Overall, our results are fully consistent with other works both on anatase thin films^[^ ^10^, ^44^ ^]^ and single‐crystals.^[^ ^8^, ^9^ ^]^

**Figure 2:**
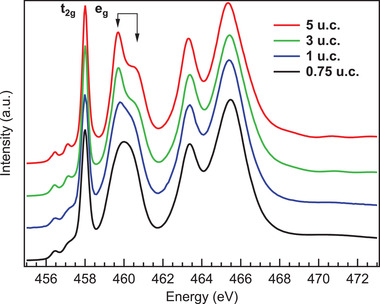
Evolution of the Ti L‐edge XAS spectrum as a function of anatase TiO_2_ film thickness, namely 5, 3, 1, and 0.75 u.c. The characteristic splitting of the e_g_ levels is marked by black arrows.

In the ultra‐thin regime, namely down to a single‐unit‐cell, the crystal structure of a film can differ from thicker samples as the chemistry, the defects or the stress occurring at the interface might stabilize other TiO_2_ allotropic forms. Knowing that the determination of the crystal structure of the thinnest samples is crucial, we have investigated the evolution of TiO_2_ anatase as a function of film thickness by means of XAS. The Ti L_2, 3_ absorption edges measured as a function of the sample thickness are presented in **Figure** **2**. Spectra display the typical shape of the Ti^4 +^ oxidation state.^[^ ^11^ ^]^ The first two peaks (458.3 and 460.5 eV) correspond to the L_3_ absorption edge, while those at 463.5 and 465.5 eV are related to the L_2_ absorption edge. One observes a further split into the *t*_2g_ and the *e*_g_ levels of the final state, as due to crystal‐field splitting.^[^ ^49^ ^]^ Moreover, a spectroscopic feature representative of TiO_2_ anatase is found at 460.5 eV (at the L_3_‐*e*_g_ peak), where additional splitting is observed, with a main peak and a shoulder at higher excitation energy. This fine structure and the ratio between the peak and the shoulder uniquely identify anatase among other TiO_2_ polymorphs (e.g., rutile).^[^ ^50^, ^51^, ^52^, ^53^ ^]^ Such observation suggests that the anatase electronic structure is conserved down to the single‐unit‐cell. In particular, the absorption spectrum of the 5 u.c. (red curve in Figure 2) has identical features of the 40 nm thick one.^[^ ^11^ ^]^ Although the double‐peak structure is clearly detectable down to 1 u.c., it transforms into a single broad peak in the case of the thinnest film (≈0.719 nm thick, i.e., at 0.75 u.c.), implying that in this last case the anatase crystal structure is not stabilized. As a matter of fact, in agreement with our previous investigation by in situ STM and ex situ HR‐TEM and low‐angle X‐ray reflectivity,^[^ ^11^, ^27^ ^]^ the TiO_2_ surface is characterized by a very low surface roughness, consistently with a pure 2D growth mode, and the 0.75 u.c. sample corresponds to a film fully covered by 0.75 u.c. of TiO_2_. Thus, a minimum of one unit cell is necessary to develop the extra‐splitting of the L_3_‐*e*_g_ absorption peak. Now we are able to set a correspondence between the anatase structure and the presence of the 2DEG.

**Figure 3:**
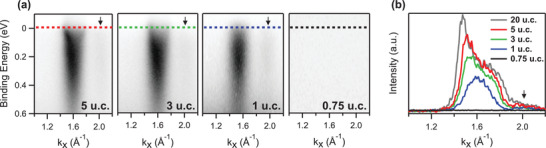
a) Evolution of the ARPES spectrum of the metallic state as a function of anatase TiO_2_ film thickness, namely 5, 3, 1, and 0.75 u.c. The spectra have been acquired at hν=46 eV around the $\bar{\Gamma }$ point of the second Brillouin zone. The dark shaded area marked by black arrows is a 2DEG replica. b) MDCs extracted at Fermi energy in correspondence with the dashed lines in panel (a). As a comparison, a MDC extracted at Fermi energy of a thick anatase TiO_2_ sample (20 u.c.) is also reported (dark grey curve).

Figure 3 reports the evolution of the 2DEG versus thickness as probed by in‐situ ARPES. We measured the 2DEG in the second BZ to avoid the undesired suppression of the photoemitted intensity caused by the strong symmetry‐related matrix element effects on the d_*xy*_ orbitals in the first BZ (Figure 1f). Panel (a) shows spectra as acquired along the surface‐projected ${\bar{\Gamma }}_{10}$–${\bar{X}}_{10}$ high‐symmetry direction on the whole sample series (i.e., 5, 3, 1, 0.75 u.c.), while panel (b) reports the momentum distribution curves (MDCs) extracted at the Fermi level. In perfect agreement with XAS data of Figure 2, the 2DEG state is visible down to 1 u.c. However, in the ultra‐thin regime it was not possible to observe quantized subbands with the same ARPES experimental resolution that allowed us to detect a second parabola in the thick TiO_2_ film (Figure 1g). The dark shaded area marked by arrows in the ARPES spectra around *k*_*x*_ = 2.0 Å^−1^ (also detectable as a bump in the MDCs of Figure 3b) is a 2DEG replica. This indicates the presence of the 4×1 reconstruction typical of the anatase (001) surface. In contrast, neither intensity nor 4×1 reconstruction are observed at Fermi energy in the film of 0.75 u.c., consistent also with the missing fingerprint of the anatase fine structure in the Ti 2p XAS. Moreover, the data set provides further insights on the depth distribution of the 2DEG within the surface region. The MDCs reported in Figure 3b reveal an increase of the electron carriers in the first few unit cells which results in a downward rigid shift of the 2DEG parabola (assuming single‐band crossing). Our results prove that no critical film thickness is required to stabilize the 2DEG at the anatase TiO_2_ surface, suggesting that the anatase 2DEG has a different nature than the 2DEG states found at the LaAlO_3_/SrTiO_3_ hetero‐interface,^[^ ^13^, ^19^, ^54^ ^]^ where a critical thickness of 1.5 nm occurs to reveal a 2DEG.^[^ ^15^ ^]^

This is best observed from the enlargement of the electron Fermi momenta *k*_F_ in Figure 3b: stark changes occur in the very first layers (i.e., from 1 to 3 u.c samples), where the left‐to‐right asymmetry in the photoemitted intensity distribution disappears for the thinner sample, consistent with the bottom of the parabola approaching the Fermi level. Accordingly, the electron density increases from n_2D_ ≈ 1.0× 10^13^ cm^−2^ (1 u.c.) to *n*_2D_ ≈ 2.3× 10^13^ cm^−2^ (3 u.c.) and *n*_2D_ ≈ 2.7× 10^13^ cm^−2^ (5 u.c.), converging to the value found previously for a thicker TiO_2_ film (≈20 nm). It is worth noting that, even in the ultra‐thin regime, the electron density is within the high carrier's concentration range reported in literature.^[^ ^8^, ^9^, ^24^ ^]^ When the film thickness increases, the 2DEG occupancy saturates, as seen by the minor changes between 3 and 5 u.c. and also between 5 and 20 u.c. samples. In fact, already the 5 u.c. film possesses the bulk‐like properties of thicker films, as expected also from the well‐defined L_3_‐*e*_g_ XAS splitting discussed above. This indicates that the 2DEG is not localized at the outermost atomic layer of the unit cell, but rather that its electronic wave‐function spans over the subsurface region, consistently with previous findings.^[^ ^23^, ^55^, ^56^ ^]^

**Figure 4:**
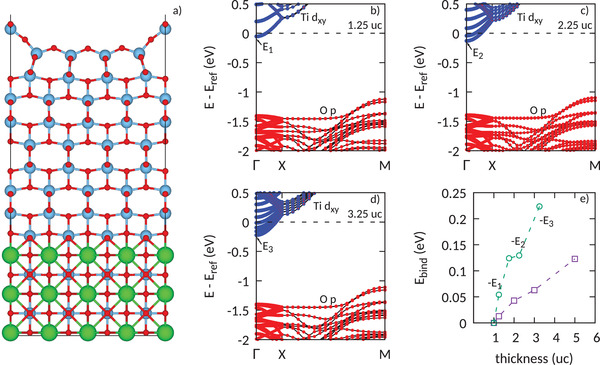
a) Atomic structure of TiO_2_ thin film: the Ti atoms are shown by means of blue spheres. The TiO_2_ surface is terminated by a fully‐relaxed 4×1 reconstruction. b–d) Electronic structures for several different TiO_2_ thicknesses. The oxygen p manifold (in red) is separated from the titanium d_*xy*_ bands (in blue). The latter disperse parabolically around the Γ point and give origin to the 2DEG. e) Evolution of the 2DEG onset as estimated by ARPES measurements (purple line) in comparison with the ab‐initio results (green line).

To explore this issue in more detail, we performed DFT‐based numerical calculations of TiO_2_ films at different thicknesses. For simplicity, we used a SrTiO_3_ substrate, since the thickness dependence of TiO_2_ low‐energy physics is not expected to depend on this specific choice. Moreover, in order to meaningfully compare the results, we aligned the substrate Sr semicore 4s states, since they are not affected by the top TiO_2_ layers, and defined our zero energy reference E_ref_ as the energy of the bottom of the Ti d_*xy*_ band at the Γ point for the 1 u.c. thick TiO_2_ film. As shown in **Figure** **4**e, our ab‐initio simulations track the evolution of the 2DEG onset. Indeed, in going from the thin limit to the thick film, the minimum of the parabolic dispersion moves downward, causing more and more electronic states to cross the Fermi level. As a direct consequence, an enlargement of the electron momenta at the Fermi level is observed, consistent with the experimental evidence of Figure 3b. While the observed experimental trend is clearly reproduced, though, the DFT calculations overestimate the binding energy by a factor of two. Speculating about the origin of these deviations, it is important to emphasize that, differently from the real TiO_2_ systems, oxygen vacancies are not considered in our calculations. The downward shift of the 2DEG onset in Figure 4b–d is solely due to the confinement of quantum‐well states (states with different *k*_*z*_ wavevector in bulk TiO_2_), while oxygen vacancies in real TiO_2_ provide the necessary electrons to pin the Fermi level inside the Ti manifold.^[^ ^8^, ^10^ ^]^

## 3 Conclusions

In summary, our photoemission results and theoretical calculation provide evidence of the presence of a 2DEG in anatase TiO_2_ down to a single‐unit‐cell. The metallic state is strictly related to the electronic structure of anatase, as revealed by the comparison between ARPES and XAS. The evolution that the 2DEG state undergoes with thickness, that is, a clear electron‐filling of the states with increasing thickness, proves extension to the subsurface region, suggesting a mechanism driven by intrinsic defects such as oxygen vacancies. In particular, we have clarified that a threshold film thickness, or critical thickness, is not necessary for the formation of a 2DEG in TiO_2_: the metallic state is established as soon as the 2D delocalization is achieved with a single‐unit‐cell. Therefore, the formation of delocalized electronic states in anatase TiO_2_ is intrinsically different from other 2DEGs observed in similar systems such as SrTiO_3_ and LaAlO_3_/SrTiO_3_ interface.

## 4 Experimental Section

### Growth

Anatase TiO_2_ thin films were epitaxially grown on (001)‐oriented LaAlO_3_ (LAO) substrates in ultra‐pure oxygen background pressure (purity 99.9999%) by pulsed laser deposition (PLD) at a dedicated vacuum chamber located at the APE‐IOM laboratory (NFFA facility, Trieste, Italy)^[^ ^11^, ^57^ ^]^ using a KrF excimer laser source at a typical energy density of about 2 J cm^−2^ and a typical laser repetition rate of 3 Hz. A stoichiometric rutile TiO_2_ single‐crystal target was used for the deposition process. The growth temperature was set to 700 °C and the oxygen background pressure to 10^−4^ mbar, while the target–substrate distance was ≈50 mm. The typical deposition rate was about 170 laser shots per TiO_2_ unit‐cell, corresponding to about 3.5 Å min^−1^: such a low growth‐rate has allowed a full control over the thin film thickness. In order to shed light on possible extrinsic mechanisms which might take place at film/substrate interface, TiO_2_ ultra‐thin films (i.e., from five unit cells down to sub‐unit‐cell thickness) were also grown on 5 nm thick buffer layers of both LaAlO_3_ (LAO) and LaNiO_3_ (LNO) grown on (001)‐oriented LAO substrates by pulsed laser deposition. Epitaxial strain‐less conditions were verified for these films by means of X‐ray diffraction measurements.

### Transmission Electron Microscopy

Cross‐sectional TEM samples were prepared with a conventional grinding and polishing technique followed by dimpling and milling with Ar ions with a Gatan PIPS ion mill.^[^ ^33^ ^]^ High resolution STEM experiments were performed on a Cs‐corrected JEOL ARM 200 CF operated at 200 keV. The probe convergence angle was 24 mrad and the HAADF detection angles were set to 68–185 mrad.

### X‐Ray Spectroscopy

The electronic properties and the chemical composition of TiO_2_ thick (i.e., 20 nm) films were explored directly in‐situ by measuring core level XPS at the high‐energy experimental endstation of APE beamline (APE‐HE) at Elettra synchrotron (Trieste, Italy), equipped with an Omicrion EA125 hemispherical analyzer. The XPS spectra were recorded with photon energy hν = 900 eV. The electronic properties as a function of the film thickness (down to sub‐unit‐cell thickness) were investigated by XAS. In particular, XAS experiments were performed in‐situ at APE‐HE in the total electron yield mode with the incident beam at fixed horizontal polarization and incidence angle of 45°; the drain current from a highly transparent mesh was used to normalize the measured signal with the incident photon flux. The electronic band dispersion has been probed in‐situ by ARPES experiments at the low‐energy endstation of APE beamline (APE‐LE). The chamber was equipped with a Scienta DA30 hemispherical electron energy and momentum analyzer (30° angular acceptance), which allowed to map the electronic band structure over the extended areas of the BZ without rotating the sample. The base pressure was ≈10^−10^ mbar and the samples were kept at liquid nitrogen. The photon energy was set to 46 eV with horizontal polarization and with a light incidence angle of 45°.

### DFT Calculations

To study and understand the evolution of the TiO_2_ thin films electronic properties, DFT simulations of slabs were performed with different TiO_2_ thicknesses. As an exemplary case, the TiO_2_ 2.25 u.c. structure is shown in Figure 4a. The projector augmented wave method with energy cutoff of 500 eV was used as implemented in the Vienna ab‐initio simulation package.^[^ ^58^, ^59^ ^]^ The PBE generalized gradient approximation^[^ ^60^ ^]^ was chosen to treat exchange and correlation. The TiO_2_ surface was modeled with the experimentally confirmed 4×1 surface reconstruction.^[^ ^43^ ^]^ Thus, the Brillouin Zone was sampled on a regular 3× 12 ×1 grid in order to account for this structure.

## Conflict of Interest

The authors declare no conflict of interest.

## Supporting information

Supporting Information

## Data Availability

The data that support the findings of this study are available from the corresponding author upon reasonable request.
