# Supplementary material for: Evidence of a 2D Electron Gas in a Single‐Unit‐Cell of Anatase TiO2 (001)
Source: Adv Sci (Weinh). 2022 Apr 5;9(16):2105114. doi: 10.1002/advs.202105114 (PMC9165519; doi:10.1002/advs.202105114)
Supplement: Supplementary file 1 — Supporting Information [file ADVS-9-2105114-s001.pdf]

Supporting Information

Evidence of a two-dimensional electron gas in a single-unit-cell of anatase TiO_2 (001)

Alessandro Troglia[1,2], Chiara Bigi[1,2], Ivana Vobornik[1], Jun Fujii[1], Daniel Knez[1], Regina Ciancio[1], Goran Drazic[4], Marius Fuchs[5], Domenico Di Sante[6,7], Giorgio Sangiovanni[5], Giorgio Rossi[1,2], Pasquale Orgiani[1,3,], Giancarlo Panaccione[1,]**

[1] Istituto Officina dei Materiali (IOM)-CNR, Laboratorio TASC in Area Science Park, S.S. 14 Km 163.5, 34149 Trieste, Italy.

[2] Dipartimento di Fisica, Università di Milano, Via Celoria 16, 20133 Milano, Italy.

[3] CNR-SPIN, UOS Salerno, 84084 Fisciano, Italy.

[4] Department of Materials Chemistry, National Institute of Chemistry, Hajdrihova 19, 1001 Ljubljana, Slovenia.

[5] Institut für Theoretische Physik und Astrophysik and Würzburg-Dresden Cluster of Excellence ct.qmat, Universität Würzburg, 97074 Würzburg, Germany.

[6] Department of Physics and Astronomy, University of Bologna, 40127 Bologna, Italy.

[7] Center for Computational Quantum Physics, Flatiron Institute, 162 5th Avenue, New York, New York 10010, USA.

* Email address: orgiani@iom.cnr.it, panaccione@iom.cnr.it.

The crystallographic properties of both $LaNiO_3$ (LNO) and TiO_2/LNO samples grown on $LaAlO_3$ substrates were explored by means of X-Ray Diffraction (XRD). Structural characterization was carried out using a four-circle diffractometer with a Cu Kα radiation source. For the LNO films, in order to have a significant signal-to-noise ratio, XRD characterization was carried out on 20 nm thick LNO sample. To evaluate both the LNO in-plane lattice parameters, reciprocal space map around the (0-13) asymmetric Bragg reflections were performed (Figure S1.a). The perfect alignment of the diffraction peaks along the Q_x-direction proves the full in-plane match between the LNO film (at least up to 20 nm in thickness) and the LAO substrate. Finally, XRD analysis was also performed on 15 nm thick anatase TiO_2 sample grown on 5 nm thick LNO buffer layered LAO substrate (Figure S1.b). As expected by the very low lattice mismatch with the LAO substrate (0.1%), preserved by the epitaxial growth of the LNO buffer layer, the anatase TiO_2 thin films grow fully matching with the in-plane lattice parameters of the LAO substrate as shown by reciprocal space map

around the (113) LAO and (116) TiO_2 asymmetric reflections showing a perfect alignment of the diffraction peaks along the Q_x-direction.

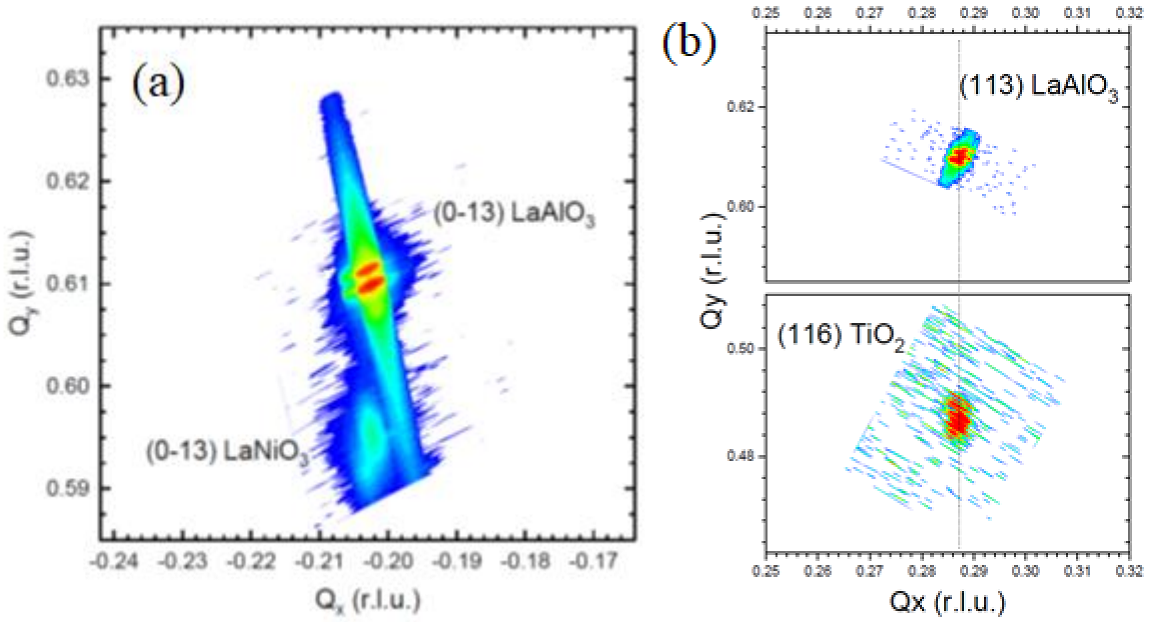

Figure S1 – (a) Reciprocal space map of a 20 nm thick LNO film grown on LAO substrates around the (0-13) asymmetric Bragg reflections; (b) Reciprocal space map of a 15 nm thick TiO_2 sample grown on a 5 nm thick LNO buffered LAO substrate around the (113) LAO and (116) TiO_2 asymmetric Bragg reflections.
